# Validation of three pain scales among adult postoperative patients in Ghana

**DOI:** 10.1186/s12912-015-0094-6

**Published:** 2015-08-11

**Authors:** Lydia Aziato, Florence Dedey, Kissinger Marfo, James Avoka Asamani, Joe Nat A. Clegg-Lamptey

**Affiliations:** Department of Adult Health, School of Nursing, University of Ghana, Legon, Accra, Ghana; Department of Surgery, University of Ghana Medical School, Accra, Ghana; Public Health Unit (Biostatistics), Korle-Bu Teaching Hospital, Accra, Ghana; Human Resource Planning and Research, Ghana Health Service, Accra, Ghana; School of Nursing, College of Health Sciences, University of Ghana, P.O. Box LG 43, Legon, Accra, Ghana

## Abstract

**Background:**

Pain assessment is an important component of pain management and health professionals require valid tools to assess pain to guide their pain management decisions. The study sought to select, develop, and validate context-appropriate unidimensional pain scales for pain assessment among adult post-operative patients.

**Methods:**

A mixed methods design was adopted. The study was conducted at two hospitals in Accra, Ghana. The qualitative phase involved 17 patients and 25 nurses, and the quantitative phase involved 150 post-operative patients. Qualitative data was collected iteratively through individual interviews and focus groups.

**Results:**

Two existing pain scales (0–10 Numeric Rating Scale [NRS] and Wong-Baker FACES [FPS] scales) and one new pain scale (Colour-Circle Pain Scale–[CCPS]) were validated. The psychometric properties of the three scales were assessed when patients had fully recovered from anesthesia. The CCPS had higher scale preference than NRS and FPS. Convergent validity was very good and significant (0.70–0.75). Inter-rater reliability was high (0.923–0.928) and all the scales were sensitive to change in the intensity or level of pain experienced before and after analgesia such as paracetamol and diclofenac suppositories, injectable pethidine, and oral tramadol had been administered.

**Conclusion:**

Using a valid tool for pain assessment gives the clinician an objective criterion for pain management. Due to the subjective nature of pain, consideration of socio-cultural factors for the particular context ensures that the appropriate tool is used.

## Background

Pain is a subjective phenomenon, and its expression and management is influenced by the culture of the individual due to the socialization process [1–3]. Context-appropriate pain assessment tools are therefore necessary for different groups of people. The lack of appreciation of a people’s culture could result in the use of pain assessment tools and techniques that are ineffective and culturally inappropriate [4]. Hence, appropriate pain assessment tools should be identified for specific cultural groups to ensure accurate pain assessment and management because every patient has the right to effective pain management [1]. Assessing pain in a culturally competent manner decreases health care disparity. This is crucial in the management of patients as pain is what brings many patients to the hospital [1].

Over the years, pain assessment tools have been validated among various populations globally [5–7]. However, in Ghana, pain assessment is not formalized within the Ghanaian clinical context and pain scales are not used for pain assessment. Indeed, previous studies indicate that post-operative pain (POP) is not managed effectively in other countries [8, 9]. In Ghana, the incidence of moderate to severe post-operative pain is about 70 % [10]. Pain assessment tools help health professionals to quantify a subjective phenomenon into objective terms to inform and evaluate pain management interventions. There are several pain assessment tools or pain scales that have been validated over the years for use by health professionals. They include the Visual Analog Scale (VAS), Verbal Descriptor Scale (VDS), Numeric Rating Scale (NRS), Faces Pain Scale and Faces Pain Scale-Revised (FPS-R) [5, 7].

The development of context appropriate pain scales is done with the active participation of users of the scales. An iterative inductive process ensures that participants make input at every stage of the development of the scale. The literature so far indicates that only the Twi or Ghanaian version of the VAS has been developed and validated in Ghana among Twi speaking post-operative gynecological patients. The original VAS was also validated in the same study [11]. However, the scale has not been introduced in clinical practice for reasons yet to be investigated. No other pain scale has been developed or validated in Ghana. Hence, pain scales are not used in the Ghanaian clinical practice. This study sought to select or develop a simple pain scale from participants’ perspectives, ideas, or previous knowledge and exposure to pain scales. Thus, no pain scale was predetermined for inclusion in the study.

This study had two phases. Phase One, the qualitative phase, was to select and develop appropriate scales for pain intensity assessment. Phase Two, the quantitative phase, sought to validate or assess the psychometric properties of the pain scales that emerged from phase one, among adult post-operative patients. The hypotheses for the validation of the pain scales were:

1. To demonstrate convergent validity, there will be a significant positive correlation between the scores of the pain scales.
2. There will be a high inter-rater consistency between pain scores of two different assessors.
3. Pain scores before analgesia will significantly differ from pain scores after analgesia (demonstrating the scales’ sensitivity to change).

## Methods

### Design

The study employed a multi-phase approach to achieve its purpose. An inductive iterative qualitative process was used to select and develop appropriate scales for pain assessment. The qualitative phase adopted an exploratory descriptive method which allowed identification of features of pain scales and development of a scale which were consistent with the culture of the participants in the study. The philosophy of the qualitative approach was that the perspectives, preferences and interpretations of individuals within a specific socio-cultural context differ [12] and should be incorporated in the development of pain scales. The quantitative approach was used to assess the psychometric properties of the pain scales.

### Setting

The first phase of the study was conducted at a tertiary health facility (Korle Bu Teaching Hospital) and a regional hospital (Ridge Hospital) in Accra, Ghana. The second phase was done at the tertiary health facility. The two hospitals have facilities for surgery where various types of surgical procedures are undertaken.

### Ethics

The study was approved by the Institutional Review Board of the Noguchi Memorial Institute for Medical Research. Permission was obtained from the authorities of the two hospitals, and individual informed consent obtained from participants both orally and written depending on the participants’ preference and educational background. Anonymity and confidentiality were ensured and participants were assigned identification codes. Identification codes were used to represent the participants: P1-P17 for patients and N1 to N25 for nurses in Phase One and A1-A150 to represent patients in Phase Two.

### Sample, procedures and data analysis for the qualitative phase

Nurses and post-operative patients on the surgical wards participated in the study. The inclusion criteria were nurses with a minimum of 6 months working experience on the surgical ward and ambulant patients on the 5th–7th day after surgery who were not in pain. The 6 months clinical experience was considered adequate exposure to enable nurses’ share their experiences on patients’ pain assessment. Patients in pain may have difficulty sharing their experiences because of the pain. Patients and nurses who did not meet the inclusion criteria or give informed consent were excluded.

Purposive sampling technique was employed to recruit participants who met the criteria. The qualitative phase involved individual interviews and focus group discussions. Individual interviews and focus group discussions were used to ensure that ideas for context appropriate pain scale features were generated and discussed from a variety of sources. In contrast to individual interviews, focus groups permitted participants to discuss pain scales. Individual in-depth interviews and focus groups were conducted iteratively to develop and select pain scales. Interviews and discussions were done at a place and time convenient for participants. The participants were allowed to express themselves in the language they were fluent in. Thus, the patients’ interviews were conducted in the local Twi dialect and the nurses’ in English. The interviews and discussions lasted 30 to 45 min. The interviews were recorded and transcribed, while notes were taken during focus group discussions. The first author who is fluent in English and Twi conducted the interviews and moderated the focus groups, and a research assistant took detailed notes during the discussions. This ensured consistency in the data collection process.

The individual interviews involved 18 participants (7 patients and 11 nurses) to generate features of appropriate pain scales. Interviews stopped when data was saturated. During the individual interviews, pain assessment of participants, use of pain scales, and features of pain scales in relation to the context of the study were explored. These were probed in relation to the culture of the patients and nurses. The ideas identified through concurrent content analysis specific to pain scales were discussed during the focus groups. The features generated that were consistent with existing scales, such as numbers and faces pain scales, were selected for discussion during the focus groups. Thus different formats of existing scales were presented and discussed.

In addition, the new culturally specific pain scale features that emerged were developed into scales with appropriate English descriptors for focus group discussions to ensure that the scale and descriptors were consistent with the cultural expression of pain. Participants were actively involved with the choice of English descriptors of the new scale and also discussed those of the existing scales. A research assistant developed all the scales in this study. None of the scales were translated because the primary focus and design of this study was not to translate existing pain scales. We aimed to select, develop and assess the psychometric properties of context appropriate pain scales. The authors are conscious of this limitation and suggest future studies could translate the scales validated in this study using the common local languages in Ghana such as Twi, Ga and Ewe.

Four focus groups were conducted iteratively to develop the new pain scales. The focus groups were made up of two nurses and two patients groups. The number of participants in each nurses’ group was 5 to 7 and consisted of both sexes. The patients’ groups were made up of 5 participants each. Detailed notes were taken during the discussions on recommendations for refinement of the scales. The decision to discard a scale was thus reached by consensus and not by the research team. Different participants were used for the interviews and the discussions to avoid participant fatigue and bias in the development of the scale.

### Data management and analysis

Qualitative data was transcribed and read several times to identify the participants’ views on features of pain scales through coding. The notes taken during the focus group discussions were also coded to ensure that any new features of pain scales were incorporated in the analysis. Concurrent content analysis and the iterative process was used to select and develop the appropriate scales. The features identified that were similar to existing scales guided the selection of two existing scales for validation in Phase Two. The data was managed with the NVivo software version 9.

Sample, procedures and data analysis for the quantitative phase: The validation and data collection lasted a period of 4 weeks during which post-operative patients on the surgical and maternity wards of the Korle-bu Teaching Hospital participated. The hospital’s average monthly surgeries of 454 (general surgeries and caesarian sections) was used as the accessible population. With a criterion level of 0.05, we used the Yamane (1967) simplified sample size formula to determine the appropriate sample size of 212. However, 150 patients (70.75 %) met the inclusion criteria during the period of data collection. In the domain of validation or assessing the psychometric properties of pain scales, previous studies have demonstrated that a sample of 150 is acceptable and the type of analysis employed is also acceptable [6, 13, 14]. A census approach was used to recruit participants who met the inclusion criteria for pain assessment. During the clinical validation phase, pain was assessed among post-operative general surgical patients and those who had caesarian sections using three pain scales (0–10 NRS; FPS; and CCPS). Two research assistants (RA1 and RA2) assessed pain with three scales in random order as follows:

1. Inter-rater reliability was assessed at 5 to 10 min intervals between RA1 and RA2 to demonstrate that if different practitioners administered the scales to the same patient or patients with similar pain intensity, consistent results will be obtained
2. Sensitivity to change was assessed by administering the pain scale pre-analgesia and 30 min post-analgesia. Analgesia administration did not take the form of experiment (treatment group and control group) because patients were given different analgesics as prescribed on the ward.
3. Convergent validity was assessed on post-operative days 1, 2 and 3. There was a single assessment at the same time each day because it was assumed that post-operative pain decreased daily after surgery.

Scale preference was assessed by ordering scales–patients indicated their preferred pain scale for assessment in order of preference, after the final pain assessment on post-operative day 3. The assessment criteria were consistent with similar psychometric assessment studies such as the time interval of assessment [15, 16]. It was assumed that changes in pain levels within 5 to 10 min of assessment would not be significant to confound the findings. Participants were shown the scales and were asked to indicate their pain levels. This was done in English or Twi. Twi is the most common local language within the context of study. The two research assistants were both fluent in Twi and were trained by an expert in the language to maintain consistency in administering the scales to patients. The Twi words for pain, translated in Ghana by previous authors [11], were applied in the process. Patients indicated their pain levels orally and the assessors recorded these in English. No observation of pain behaviour was collected in this study.

Statistical analysis was performed using statistical package for the social sciences (SPSS) version 22 (SPSS. Chicago, Illinois, United States of America [USA]). Descriptive statistics were presented in mean and standard deviation (SD), frequency and percentage. Means were compared between groups using Student *t* test and one-way analysis of variance (ANOVA) for normally distributed variables and Kruskal Wallis was used for non-normally distributed variables. Proportions were compared using Chi-square and Fisher exact test, in case of small cells. Bonferroni test was used to adjust for alpha for multiple pair wise comparison. *P*-value <0 0.05 was considered statistically significant.

### We assessed the following psychometric properties of the scales

Convergent validity by Pearson’s correlation coefficient, inter-rater reliability by interclass correlation coefficient (ICC) [17], sensitivity to change in pain intensity following analgesia and with increasing duration after surgery (post-operative day 1 to 3) using repeated measures of ANOVA [18].

Copyright permission was sought from Elsevier to use the Wong-baker FACES pain scale (Wong & Baker, 2001) for clinical pain assessment. The 0–10 NRS is a free access pain scale. Identification codes used for scales were: Scale 1 = 0–10 NRS (S1); Scale 2 = Wong-Baker Faces Pain Scale (S2); Scale 3 = Colour-Circle pain Scale (S3).

### Trustworthiness for qualitative phase

Processes undertaken to ensure rigor or validity and reliability included effective probing to ensure that participants’ ideas on features of pain were fully explored. Moderation of focus group discussions and interviews were conducted by the first author who is experienced in qualitative data collection. Concurrent and iterative data collection and analysis were done to ensure identification and development of pain scales that were appropriate for the context of the study. All the authors were involved in data verification and analysis to ensure that participants’ views were captured. Detailed audit trail was maintained so that future researchers can confirm the processes used in this study.

### Validity and reliability for quantitative phase

Patients who were involved in the scale development and selection did not participate in the clinical validation of the scales as they had been discharged at the time of validation. Also, two research assistants assessed pain for all the patients in this study to avoid participant familiarity, which could confound the data generated especially where the assessor demonstrated negative attitude during pain assessment. Quantitative data was checked for completeness and omissions before analysis to avoid error in analysis.

## Results

The results of this study are presented in two sections; Section One presents the tool development process (qualitative) and Section Two reports the psychometric properties (quantitative) findings.

The development and selection of the pain scales involved 25 clinical nurses and 17 post-operative patients (for both interviews and focus group discussions). The nurses consisted of 12 staff nurses, 10 nursing officers, and three senior nursing officers. The 5 male and 20 female nurses who were involved in the pain scale development were between 26 and 50 years of age and had 6 to 15 years experience in surgical nursing. With regard to education, four nurses had nursing certificates, 17 had diplomas, and four held bachelor degrees in nursing. The 17 post-operative patients consisted of seven males and 10 females between the ages of 25 to 45 years. Ethnic breakdown of these participants were as follows: Twi (22), Ewe (8), Ga (7), and Dagomba (5).

The initial interviews generated six features of potential pain scales (faces, fingers, colours, squares, numbers, and plain circles) and the iterative data collection and analysis process further refined and reduced the number of scales to the three scales validated. Features of pain scales identified in the interviews guided selection of existing scales that was consistent with the data (FPS and NRS). Also, new ideas were developed according to participants’ suggestions using a 6-point scale (CCPS). A research assistant developed all the potential new pain scales. All the scales were subjected to focus group discussions iteratively. Sample interview data from which potential pain scales were generated is presented in Table 1.

**Table 1:** Interview data identifying pain scale feature

Participant	Participants’ quote	Pain scale feature
P1	*I really squeezed my face*	Faces
P2	*I cry because of the pain …when there is no pain, I do not cry. Patients can use FACES scale if they have not been to school*	
N11		
N1	*I used my fingers for the patient to indicate the number of fingers and the pain level.*	Fingers with words
N6	*…So you would say the thumb is very severe and the little finger is no pain*	
N2	*I think maybe different colours can be used from no pain to very, very severe pain like white, green, yellow, blue, red and black in that order, he will be able to point to the colour representing the level of pain; …the very, very severe one can be black.*	Colours with words
N3	*…red will be for most severe because if you go around, red is mostly used for danger. So if you see red here it means there is something dangerous here so it alerts you*	
N2	*…can also use different sizes of square boxes so patients can choose one*	Squares with words
N3	*I know about pain assessment, using the scale from 0 to 10, I show it to them and we usually do not do it anyway but if I am to do that, I will show the scale to them as 0 no pain at all 1, then it increases up to 10 and that being the most severe. So depending on how severe they think their pain is then they will choose one of the numbers then they will tell me which it is.*	Numbers (0–10)
N7	*…we draw circles with smallest representing, no pain in that order*	Plain circles with words

**Fig. 1:**
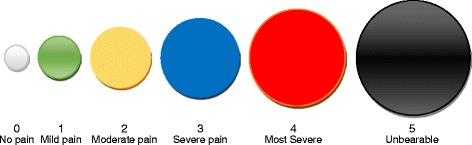
Colour-circle pain scale

**Fig. 2:**
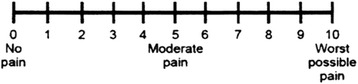
0–10 numeric rating scale

**Fig. 3:**
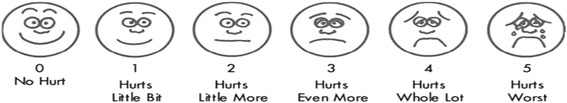
Wong-Baker FACES pain scale

Iterative focus groups indicated that focus groups One and Two discussed the 6 scales and participants preferred four scales (NRS, FPS, plain circle, and colour scales). Focus groups Three and Four discussed the four scales and further merged plain circle and the colour circle into one new scale (Colour-Circle Pain Scale) (Fig. 1). The scales that were not selected were discarded (finger, and square scales). The two existing scales (0–10 NRS and FPS) (Figs. 2 and 3 respectively) remained unchanged.

The colour-circle pain scale (Fig. 1) was developed as follows:

*Six plain circles each of 1″ height and width, were inserted from auto shapes in Microsoft Word, onto the page. The circles starting from a size of 0.4″ in diameter were increased in sizes of 0.3″, until the 6th circle. This was aimed at differentiating the various levels of pain. Each circle was filled with different colours, with their borders having the same colours, to differentiate between the various pain levels, hence the entire pain scale comprises the following colours sequentially: 1–Linear Up Gradient–Dark; 2–Horizontal Gradient–Accent 6; 3–Gold Accent 4, Lighter 60 %; 4–Blue; 5–Red; and 6–Horizontal Gradient–Dark. All drawings were grouped together with their captions for consistency and uniformity of interpretation.*

All the 3 scales that emerged had word descriptors and numbers to ensure uniformity in interpretation and documentation of findings.

In Phase Two, 150 surgical patients with a mean age of 33.1 years (SD 12.2) were enrolled in the study. Approximately 77 % of the patients were females, with 33 % of them less than 30 years old. Most (66.0 %) of the patients were post caesarian, 15.33 % post laparotomy and 4.67 % had lower limb amputation. Out of the male patients, 40 % had laparotomy. Majority (48.0 %) of the patients were of the Twi ethnic background and the least (6.0 %) were from the Dagomba ethnic background (Table 2).

**Table 2:** Patient Socio-Demographic characteristics

Varaibles		Age group			Total
		<30	30–39	40+	
Gender	Male	11.33 % (17)	2.67 % (4)	9.33 % (14)	23.33 % (35)
	Female	33.33 % (50)	32.00 % (48)	11.33 % (17)	76.67 % (115)
	Total	44.67 % (67)	34.67 % (52)	20.67 % (31)	100.00 % (150)
Ethnicity	Akan	22.67 % (34)	16.00 % (24)	9.33 % (14)	48.00 % (72)
	Dagomba	2.00 % (3)	2.67 % (4)	1.33 % (2)	6.00 % (9)
	Ewe	5.33 % (8)	4.00 % (6)	4.67 % (7)	14.00 % (21)
	Fante	1.33 % (2)	2.67 % (4)	1.33 % (2)	5.33 % (8)
	Ga	13.33 % (20)	9.33 %(14)	4.00 % (6)	26.67 % (40)
	Total	44.67 % (67)	34.67 % (52)	20.67 % (31)	100.00 % (150)
Type of Surgery	Caesarean section	30.00 % (45)	30.00 % (45)	6.00 % (45)	66.00 % (99)
	Amputation of leg	1.33 % (2)	0.00 % (0)	3.33 % (5)	4.67 % (7)
	Laminectomy	2.67 % (4)	1.33 % (2)	4.00 % (6)	8.00 % (12)
	Laparotomy	7.33 % (11)	1.33 % (2)	6.67 % (10)	15.33 % (23)
	Others ^a^	3.33 % (5)	2.00 % (3)	0.67 % (1)	6.00 % (9)
	Total	44.67 % (67)	34.67 % (52)	20.67 % (31)	100.00 %
Language of Administration	English	24.67 % (37)	13.33 % (20)	2.67 % (4)	40.67 % (61)
	Twi	20.00 %(30)	21.33 % (32)	18.00 % (27)	59.33 % (89)
	Total	44.67 %(67)	34.67 % (52)	20.67 % (31)	100.00 % (150)

^a^Others include Craniotomy, Thyroidectomy, Excision of bladder tumour and Right gluteal sinus

### Convergent validity

As shown in Table 3, convergent validity of the three scales was very good with significant Pearson’s correlation coefficients ranging from 0.70 to 0.75, *P* = 0.01). The hypothesis that there will be a significant correlation between the scores of the pain scales (i.e. Hypothesis One) was therefore supported.

**Table 3:** Convergent validity: Pearson correlation coefficients between the three pain scale in all the subjects

		Scales		Level of convergent validity
		S1	S2	
RA1	S1			
	S2	0.707**		Very good
	S3	0.750**	0.749**	Very good
RA2	S1			
	S2	0.786**		Very good
	S3	0.757**	0.738**	Very good

**P-value <0.01

### Inter-rater reliability

We used interclass correlation coefficient (ICC) to assess the inter-rater reliability of the scales. Interclass correlation of the scales was high (range 0.92–0.93) indicating a very high level of agreement between scores from two raters (RA1 and RA2), supporting Hypothesis Two. The interclass correlation coefficient for S1 was 0.92 which indicated a high level of reliability. Eighty percent (120) of the patients had identical readings, 16 % (24) had readings with discrepancy of 1.0, 2.3 % (5) had readings with discrepancy of 2 and only one patient had readings with discrepancy of 5.

The interclass correlation coefficient for S2 was 0.93, also indicating a high level of inter-rater reliability. The mean difference between the two readings was 0.05; the difference between the two readings was as large as 3.5 % of mean readings. The patients that had identical readings were 88.0 % (132), discrepancy in readings of 1.0 was 10 % (15) and discrepancy in readings of 2.0 was 2 % (3).

The interclass correlation coefficient for S3 was 0.93. The mean difference between the two readings was 0.07 and the difference between the two readings as large as 4 % of mean readings. Out of the 150 patients, 92 % (138) had identical readings, 6.6 % (10) had discrepancies in readings of 1 and 1.3 % (2) had discrepancies in the reading of 2. In addition, we found no difference of inter-rater reliability of scales among the age groups (S1: F(254.4,0.42) = 0.122, *P* = 0.886; S2: F(240.3, 2.07) = 0.633, *P* = 0.532; S3 F(272.1, 1.07) = 0.289, *P* = 0.749). These findings demonstrate that if different practitioners administered the scales to the same patient or patients with similar pain intensities, similar or consistent results would be obtained.

### Sensitivity to change

Sensitivity was assessed for all three scales by measuring change in pain intensity before and after analgesia administration (see Table 4) and rate of change of effect size over post-operative days (see Table 5). There was a significant decrease in pain score in all the three scales when comparing the pre and post analgesia test. Mean differences were S1: 2.3(2.1–2.5); S2: 1.5(1.4–1.6); and S3: 1.4(1.3–1.5). This indicates that all three scales were sensitive to changes in pain levels before and after analgesia. However the sensitivity of the three scales was the same among the age groups. At a 95 % confidence level, Hypothesis Three was supported (*P* = 0.001).

**Table 4:** Rate of change of effect size over the post-operative days

Scales	Post-operative days		
	1st–2nd	1st–3rd	2nd–3rd
S1	1.9*	3.8*	1.8*
S2	1.2*	2.1*	1.0*
S3	1.1*	2.0*	0.8*

*P < 0.001 adjusted for Bonferroni correction

**Table 5:** Sensitivity of scale to analgesia

Scales	Pretest	Posttest	Mean difference (95%CL)	T	*P*-value
	*N* = 150	*N* = 150	Pretest-Posttest		
S1	7.2 ± 1.4	4.9 ± 1.3	2.3 (2.1–2.5)	24.5	<0.001
S2	3.3 ± 1.1	1.79 ± 0.7	1.5 (1.4–1.6)	22.7	<0.001
S3	3.1 ± 0.9	1.73 ± 0.7	1.4 (1.3–1.5)	24.0	<0.001

S1 and S2 recorded significant differences in the level of pain between patients who required pethidine and those who required either diclofenac / paracetamol suppository or oral paracetamol. No significant difference in pain levels was observed among patients who required the non-narcotic analgesics diclofenac and paracetamol. Also, S3 recorded significant differences in the level of pain between patients who required pethidine and those who required paracetamol tablets (Table 6).

**Table 6:** Post hoc analysis of the source of variation in pretest pain levels

Pre-test	Name of analgesic given	Supp Diclofenac	Supp Paracetamol	Tablet Paracetamol
S1	IM Pethidine	0.987*	1.970*	2.750*
	Supp Diclofenac	-	0.983	1.763
	Supp Paracetamol	-		0.780
	Tablet Paracetamol	-	-	-
S2	IM Pethidine	0.314	0.882*	0.972*
	Supp Diclofenac	-	0.568	0.658
	Supp Paracetamol	-	-	0.090
	Tablet Paracetamol	-	-	-
S3	IM Pethidine	0.380	0.592	1.222*
	Supp Diclofenac	-	0.212	0.842
	Supp Paracetamol	-	-	0.630
	Supp Paracetamol			

* The mean difference is significant at 0.05 level

### Scale preference

We assessed the scale preference of the three pain scales. A third of the patients preferred all the three scales (S1, S2, and S3) and in descending order, the preference of the scales among the other two thirds was S3, S2, and S1. There was significant variation of preference among the age groups (*X*2 = 13.19, df = 2, *P* = 0.035), older (40 years and above) patients (51.6 %) had more preference for S3 while the patients less than 40 years showed no specific preference (Table 7).

**Table 7:** Preference of the three pain scales

Scales	Level of preference		
	1st	2nd	3rd
S1	13.3 %(20)	14.0 %(21)	44.7 %(67)
S2	22.0 %(33)	40.7 %(61)	9.3 %(14)
S3	36.7 %(55)	17.3 %(26)	18.0 %(27)
Preferred all equally	28.0 %(42)		

## Discussion

The participatory and inductive approach adopted in this study is consistent with approaches adopted by previous researchers in developing scales [19, 20]. This approach was useful in developing a context appropriate simple pain scale for pain assessment. The subjective nature of pain supports the participatory approach employed to develop the new pain scale. This is evident in the findings from the second phase where scale preference was assessed and indicated significant preference for the colour-circle pain scale (S3). The high preference of the colour-circle pain scale may be attributed to its cultural significance or relationship. Majority of participants were Twi and Ga; and the colours could be of significance to these ethnic groups.

Different colours have various meanings among different ethnic groups in Ghana. Red and black are used for funerals or are associated with death and mourning and red represents danger especially among the Twi and Ga people who formed the majority in this study. The other colours used for the scale have been associated with the following in Ghana: blue represents tenderness or love, yellow shows royalty or glory, green means newness or fertility and white means purity or joy [21]. In this study, the cultural meanings of these colours were not explored and the scale was developed from suggestions of participants as representations that they could relate to different pain intensities. Thus, the verbal descriptors that were associated with the colours were inductively developed in this study. Therefore, future studies could investigate and validate the CCPS among different ethnic groups in Ghana to further generalize its appropriateness within the socio-cultural context of Ghana.

There seems to be global agreement that red represents severe pain as used in other pain scales [16, 22]. However, this study adds a dimension of increasing circles. The findings of this study indicate the need for a more elaborate study delineating the red and black colour representing unbearable pain among other ethnic groups in Ghana to confirm which of the two colours represents the most severe pain appropriately. Also, existing colour scales and the CCPS could be discussed and validated in Ghana to establish any variations among the scales. It is noted that colour pain scales are used predominantly among children such as the colour FACES pain scale.

Nurses are admonished to understand the patient’s socio-cultural context in order to use the appropriate pain assessment tools and pain management techniques [4]. The nurse performs pain assessment and patients’ self-report of pain is considered the most accurate [23]. The clinical nurses in this study, being Ghanaians, understand the culture of their patients and could contribute to develop and refine the appropriate scale that fits their clinical practice and suits the patients they care for. Illiteracy rate in Ghana is high [24] and patients who can not read and write or conceptualize pain in numerical terms could use the FPS or CCPS to report pain. Nurses within the context of the study do not routinely use any validated pain scales [8]. Context appropriate pain scales are thus more likely to be used.

It is noted that the assessment of validity and reliability of pain scales among a population include the assessment of scale preference, content validity, convergent validity, construct validity, inter-rater reliability, and sensitivity to change depending on the type of pain scale [25–27]. Hence, the psychometric assessments employed in this study are consistent with previous studies. For the interpretation of validity and reliability coefficient, we used the following criteria: Pearson’s correlation >0.70 considered as high relation with very good level of reliability and validity, 0.40–0.69 considered as moderate relation with good level of reliability and validity, 0.20–0.39 considered as low relation with fair level of reliability and validity, and <0.20 considered as very low relation with poor reliability and validity [28]. For the interclass correlation we used the conventional coefficient interpretations: values of 0.40–0.75 are fair to good and values higher than 0.75 are excellent [29].

The ability of patients to report pain using the three pain scales supports previous observation that pain assessment involves an estimation of pain intensity according to pre-determined objective criteria such as the use of pain scales [30]. However, comprehensive pain assessment involves assessment of components of pain such as location, intensity, quality, onset, duration variations, rhythms, manner of expressing pain, relieving and aggravating factors, and effects of pain [23]. Comprehensive pain assessment is necessary to establish diagnosis; however, amongst post-operative patients, pain mainly results from the surgical incision. The post-operative patient however might not have the energy or right level of consciousness necessary for comprehensive pain assessment due to the physiological effects of surgery and anesthesia. Subsequently, assessment of pain intensity is considered paramount within the post-operative context in this study.

The effective use of self-report pain scales hinges on adequate patient education. It has been observed that inadequate knowledge of patients on the use of pain scales could result in errors in pain assessment and this can lead to detrimental effects such as administering wrong doses of analgesics [23]. Hence, the authors believe that client education should accompany the use of appropriate scales that are essential for accurate pain assessment [7]. In view of this, health professionals such as nurses are to commit themselves to patient education on pain assessment and pain management and they should ensure that the patients understand what is communicated. The effective use of pain scales would involve the patient in planning their care since pain assessment findings would inform the type, dosage and frequency of analgesia administered.

The study was limited to adult post-operative patients, hence future studies could validate pain scales among children and those with chronic pain. Also, multidimensional pain assessment tools could be validated that afford comprehensive pain assessment. The tools can be translated into the major languages in Ghana to enhance pain assessment in clinical practice and for future pain intervention research. The CCPS was the most preferred in this study. However, patients with colour blindness or sight problems may be unable to use the CCPS without assistance.

## Conclusion

Although pain is a subjective abstract phenomenon, the use of the appropriate scale can elicit valid findings that can be used to guide health professionals in their care. The development and validation of the CCPS supports the need to consider the socio-cultural factors in the choice of appropriate assessment scales for a particular population. It would be necessary to involve the Ghana Health Service and other stakeholders to introduce formal pain assessment in Ghana based on the findings from this study.

## Abbreviations

NRS: Numeric rating Scale
FPS: Wong Baker FACES pain scale
FPS-R: Faces pain scale-revised
CCPS: Colour-circle pain scale
POP: Post-operative pain
VAS: Visual analog scale
VDS: Verbal descriptor scale
ANOVA: One-way analysis of variance
ICC: Interclass correlation
SD: Standard deviation
SPSS: Statistical package for the social sciences
USA: United States of America
